# Prehypertension in adolescents with cardiovascular risk: a comparison between type 1 diabetic patients and overweight subjects

**DOI:** 10.1186/s13104-016-1839-3

**Published:** 2016-02-24

**Authors:** Valentina Giacchi, Tiziana Timpanaro, Donatella Lo Presti, Stefano Passanisi, Carmine Mattia, Pasqua Betta, Chiara Grasso, Manuela Caruso, Pietro Sciacca

**Affiliations:** Department of Pediatrics, AOU Policlinico-Vittorio Emanuele, via Santa Sofia 78, 95123 Catania, Italy; Pediatric Endocrinology, AOU Policlinico-Vittorio Emanuele, via Santa Sofia 78, 95123 Catania, Italy; Pediatric Cardiology, AOU Policlinico-Vittorio Emanuele, via Santa Sofia 78, 95123 Catania, Italy

**Keywords:** Ambulatory blood pressure measurements, Cardiovascular risk, Type 1 diabetes patients, Overweight subjects

## Abstract

**Background:**

Adolescents with type 1 diabetes and obesity present higher cardiovascular risk and ambulatory blood pressure measurements (ABPM) has been shown to predict vascular events, especially by identifying the nondipper status. The aim of our observational cross-sectional study conducted in adolescents with type 1 diabetes, overweight subjects and healthy controls was to assess mean blood pressure parameters to identify subclinical cardiovascular risk.

**Methods:**

The study included adolescents patients with type 1 diabetes followed in our Pediatric Department in University of Catania between January 2011 and 2013. A total of 60 patients were enrolled, and 48 (32 male and 16 female) completed the study. For each subject we performed systolic and diastolic Ambulatory Blood Pressure Measurements (ABPM) during wakefulness and sleep recording blood pressure every 30 min for 24 h with the Tonoport V/2 GE CardioSoft V6.51 device. We compared the data of patients with those of overweight subjects and healthy controls.

**Results:**

ABPM revealed no significant difference between type 1 diabetic patients and overweight subjects in 24 h Systolic, 24 h Diastolic, Day-time Systolic, Night-time systolic and Day-time Diastolic blood pressure values but significantly different values in Night-time Diastolic blood pressure values (p < 0.001). We found significant differences between type 1 diabetic patients and healthy controls in all 24 h Systolic (p < 0.001), 24 h Diastolic (p < 0.01), Day-time Systolic (p < 0.01), Night-time Systolic (p < 0.001), Day-time Diastolic (p < 0.05) and Night-time Diastolic (p < 0.001) blood pressure values. We detected hypertension in 12/48 (25 %) type 1 diabetic patients and in 10/48 overweight subjects (p = 0.62; OR 1.2; CI 0.48–3.29), whereas no-one of healthy controls presented hypertension (p < 0.001). We observed nondipper pattern in 40/48 (83.3 %) type 1 diabetic patients, in 33/48 (68.8 %) overweight subjects (p = 0.094; OR 2.27; CI 0.85–6.01), and in 16/48 (33.3 %) of healthy controls (p < 0.001; OR 10; CI 3.79–26.3).

**Conclusions:**

ABPM studies might help to define a subset of patients at increased risk for the development of hypertension. In evaluating blood pressure in type 1 diabetes and overweight subjects, ABPM should be used since a reduced dipping can indicate incipient hypertension.

## Background

According to the World Health Organization (WHO) cardiovascular disease is the number one cause of death globally: more people die annually from cardiovascular disorders than from any other cause [1].

In recent years hypertension has shown an increase in the prevalence among adolescents and type 1 diabetes and obesity are two conditions with higher risk [2].

Type 1 diabetes is associated with a higher cardiovascular morbidity and mortality rate and hypertension is one of the risk factors leading to increased incidence of vascular events diabetic subjects [3].

Obesity is another independent risk factor for hypertension and more than 60 % of hypertensive patients are overweight [4].

Ambulatory blood pressure measurements (ABPM) have been shown to predict vascular events more accurately than office blood pressure (BP) or random blood pressure measurements and can identify the nondipper status, characterized by a decrease in physiological night-time drop of BP, constituting the first sign of the increase of pressure load on blood vessels [5, 6].

### Aim of the study

The aim of our observational cross-sectional study, performed in adolescents patients with type 1 diabetes since at least 3 years, overweight subjects and healthy controls aged between 13 and 21 years, was to assess mean blood pressure parameters, to identify subclinic cardiovascular risk of these patients.

## Methods

The mean age of the patients was 15.4 ± 2.3 (13–21) years and duration of diabetes was 8 ± 3.2 (3–17) years.

The study included adolescent patients with type 1 diabetes followed in our Cardiology and Endocrinology Clinic of Pediatric Department in University of Catania in Italy between January 2011 and 2013. A total of 60 patients were enrolled, and 48 (32 male and 16 female) completed the study.

Every patient if of age, or at least one parent or legal guardian if underage, gave their written informed consent before the patient’s inclusion in the study. The study was conducted in accordance with the Helsinki Declaration, and the study protocol was approved by the (local) Ethics Committee of the Medical University of Catania.

The diagnosis of diabetes was made according to the current criteria of the GUIDELINES ISPAD American Diabetes Association: symptoms of diabetes; random plasma glucose or 2-hour plasma glucose during oral glucose tolerance testing of 200 mg/dL or greater, or fasting plasma glucose of 126 mg/dL or greater; detection of diabetes-specific autoantibodies; increased glycosylated hemoglobin A1c (HbA1c) levels [7].

Inclusion criteria comprised a diagnosis of type 1 diabetes for >3 years, a mean HbA1c value between 6.5 and 10.7 % for the past 12 months, Tanner stage 3 or above.

Exclusion criteria were other coexisting metabolic, endocrine, or genetic disease; taking any medications affecting substrate metabolism (excluding insulin), psychotropic medications, weight loss medications, and oral contraceptives, a cut-off HbA1c level of 10.7 % (to control for potentially independent effects of extreme hyperglycemia), prepubertal or early pubertal children (Tanner stage 1 and 2) (to control for the well-described changes in the rates of diabetic complications after puberty). Seven children with Celiac disease, four with thyroiditis and another one with vitiligo dropped out the study.

The mean age of the patients was 15.4 ± 2.3 (13–21) years and duration of diabetes was 8 ± 3.2 (3–17) years.

Scheduled examination contemplates anamnestic assessment including age, adherence to drug treatment, smoking habits, alcohol consumption, duration of diabetes, other illness associated, family history of cardiovascular diseases; clinical examination with noting the presence and degree of acanthosis nigricans if present, measurement of height and weight with calculation of body mass index (BMI), systolic and diastolic BP, ABPM; blood testing including mean value of HbA_1c_ in the last year, lipid profile and microalbuminuria.

Height was measured by stadiometer and weight was measured on a calibrated scale as part of routine clinical care. BMI was calculated as weight in kilograms divided by height for meters-squared (kg/m^2^), with percentiles calculated from the year 2000 CDC growth charts and reference datasets [8] to classify children as normal weight or underweight (BMI <85th BMI-for-age percentile); overweight (BMI 85th–94th (94.9) BMI-forage percentile); or obese (BMI ≥95th BMI-for-age percentile) [9].

We determined HbA1c with high-performance liquid chromatography and measured urinary excretion of albumin in 24-h urine samples with the nephelometric method. Total cholesterol levels were measured by standardized enzymatic colorimetric assay.

For each subject we performed systolic and diastolic ABPM during wakefulness and sleep in the non dominant arm recording blood pressure every 30 min for 24 h with the Tonoport V/2 GE Cardio Soft V6.51 device. Cuff size was selected according to the measurement of mid arm circumference. Patients were instructed to perform their usual activities, avoiding heavy physical exercise and alcohol consumption. The device was installed in the morning and the subjects were asked to document their sleeping hours and activities while awake in diaries. We examined the following data: mean systolic and diastolic BP parameters in 24 h, day-time and night-time; diurnal index (the difference between mean day-time and night-time values given as the percentage of mean day-time values), percent of hypertensive measures, and percentage of nocturnal drop of systolic and diastolic BP. We defined hypertension as 24-h systolic/diastolic/mean arterial blood pressure values over the 95th percentile based on sex and height [10] according to American Heart Association Atherosclerosis, Hypertension and Obesity in Youth Committee of the Council on Cardiovascular Disease in the Young. Patients were classified as dipper if mean systolic or mean diastolic BP decreased 10 % or more during the sleep period.

We compared the data of patients with those of overweight subjects and healthy controls. We identified overweight subjects as people with BMI over 85th for age percentile without other diseases and healthy controls as subjects with normal axiological parameters and without cardiovascular, metabolic, respiratory, neurological, gastrointestinal, blooding diseases.

### Statistical analysis

For nominal characteristics, the number of patients and percentages are given. Descriptive statistics were calculated for all demographic and clinical variables. For each data we calculated mean ± SD (Standard Deviation). For the evaluation of statistically significant parameter differences within the whole sample and specific subgroups, we used the independent two-sample Student’s *t* test. We calculated statistically significant differences for diabetic patients and overweight subjects or healthy patients using the χ^2^ test. Statistical significance was set at levels of p < 0.05, p < 0.01, and p < 0.001.

The correlation between numerical variables was calculated through Spearman’s correlation coefficient. For the categorical variables we analyzed the odds ratio (OR) with the 95 % confidence interval.

## Results

The demographic and clinical characteristics of the subjects are listed in Table 1. There was no significant difference in age and gender among type 1 diabetics patients, overweight subjects and healthy controls, as well as in BMI and total cholesterol values between diabetic patients and healthy controls; instead there were different values in BMI (p < 0.001) and total cholesterol (p < 0.01) between diabetic patients and overweight subjects. Among diabetic patients, 13/48 (27 %) presented albuminuria. We assumed normal values of HbA1c and microalbuminuria in either overweight subjects and healthy controls.

**Table 1:** Demographic and clinical characteristics of diabetic patients, overweight subjects and healthy controls

Variables	Diabetics	Overweight subjects	p value	Healthy controls	p value
Age (years) (M ± SD)	15.4 ± 2.3	14.5 ± 2.9	ns	15.2 ± 2.7	ns
Sex M/F (%)	32/16 (66.7/33.3)	33/15 (67.8/31.2)	ns	38/10 (79.1/20.9)	ns
BMI (kg/m^2^)	20.1 ± 3.8	31.9 ± 3.38	<0.001	21.3 ± 3.31	ns
Diabetes duration (years)	8 ± 3.2	–	–	–	–
HbA1c (%)	7.9 ± 1.06	4.9 ± 0.35	<0.001	4.7 ± 0.42	<0.001
Total cholesterol (mg/dL)	153.4 ± 30.3	171.9 ± 15.5	<0.01	146.9 ± 26.3	ns
Albuminuria (mg/day)	3.5 ± 7	–	–	–	–
Albuminuria (n of patients)	13 (27 %)	–	–	–	–

The blood pressure profiles and the results from analysis of OR with the 95 % confidence interval and t student test of all subjects are summarized in Tables 2, 3. ABPM revealed no significant difference between type 1 diabetic patients and overweight subjects in 24 h Systolic, 24 h Diastolic, Day-time Systolic, night-time systolic and Day-time diastolic BP values but significantly different values in night-time diastolic blood pressure values (p < 0.001). Instead we found significant difference between type 1 diabetic patients and healthy controls in all 24 h Systolic (p < 0.001), 24 h Diastolic (p < 0.01), Day-time Systolic (p < 0.01), Night-time Systolic (p < 0.001), Day-time Diastolic (p < 0.05) and Night-time Diastolic (p < 0.001) blood pressure values.

**Table 2:** Blood pressure profiles of diabetic patients, overweight subjects and healthy controls

Variables	Diabetics	Obese subjects	p value	Healthy controls	p value
24 h Systolic blood pressure (mmHg) (M ± SD)	123.9 ± 11	125.2 ± 8	ns	116.1 ± 6.5	<0.001
24 h Diastolic blood pressure (mmHg) (M ± SD)	73.2 ± 8.3	74.4 ± 8.1	ns	68.3 ± 4.6	<0.01
Day-time Systolic blood pressure (mmHg) (M ± SD)	125 ± 11.4	127.2 ± 8.4	ns	119.5 ± 6.3	<0.01
Night-time Systolic blood pressure (mmHg) (M ± SD)	120.3 ± 12.2	118.8 ± 10.8	ns	110.5 ± 8.9	<0.001
Day-time Diastolic blood pressure (mmHg) (M ± SD)	75.7 ± 9.9	76.9 ± 9.1	ns	72.3 ± 5.5	<0.05
Night-time Diastolic blood pressure (mmHg) (M ± SD)	69.1 ± 8.7	67.8 ± 8.9	<0.001	62.6 ± 5.9	<0.001
Dipper (n)	8 (16.7 %)	18 (37.5 %)	<0.05	32 (66.7 %)	<0.001
Nondipper (n)	40 (83.3 %)	33 (68.8 %)	ns	16 (33.3 %)	<0.001
Hypertension (n)	12 (25 %)	10 (20.8 %)	ns	0	<0.001
Hypertension + nondipper (n)	4 (8.3 %)	3 (6.2 %)	ns	0	<0.05

**Table 3:** Results from analysis of odds ratio (OR) with the 95 % confidence interval and t student test

	Diabetics	Obese subjects	OR	95 % CI	p	Healthy controls	OR	95 % CI	p
Nondipper (n)	40 (83.3 %)	33 (68.8 %)	2.27	0.85–6.01	ns	16 (33.3 %)	10	3.79–26.3	<0.001
Hypertension (n)	12 (25 %)	10 (20.8 %)	1.2	0.48–3.29	ns	0	–	–	<0.001
Hypertension + nondipper (n)	4 (8.3 %)	3 (6.2 %)	1.3	0.28–6.44	ns	0	–	–	<0.05

We detected hypertension in 12/48 (25 %) type 1 diabetic patients and in 10/48 overweight subjects (p = ns; OR 1.2; CI 0.48–3.29), whereas no-one of healthy controls presented hypertension (p < 0.001). We observed nondipper pattern in 40/48 (83.3 %) type 1 diabetic patients, in 33/48 (68.8 %) overweight subjects (p = ns; OR 2.27; CI 0.85–6.01), and in 16/48 (33.3 %) of healthy controls (p < 0.001; OR 10; CI 3.79–26.3). Finally we noticed hypertension and nondipper pattern at the same time in 4/48 (8.3 %) type 1 diabetic patients and in 3/48 (6.2 %) overweight subjects (p = ns; OR 1.3; CI 0.28–6.44), whereas no-one of healthy controls showed these anomalies in blood pressure values (p < 0.001). We found 8/13 (61.5 %) dipper status among patients with albuminuria and 32/35 (91.4 %) dipper status among patients without albuminuria.

We did not find correlation between alteration of blood pressure circadian rhythm and HbA1c, duration of diabetes, total cholesterol, BMI or microalbuminuria (p = ns).

## Discussion

Our study shows the relevance of borderline systemic arterial pressure alterations identifiable by using of Ambulatory blood pressure Monitoring in type 1 diabetic patients and overweight subjects in adolescence.

In our series we detected significant difference in number of hypertensive subjects between type 1 diabetic patients and healthy controls (p < 0.001), demonstrating that type 1 diabetes and obesity may be a considerable risk factor for hypertension, but not between type 1 diabetic patients and overweight subjects (p = ns) because also obesity, likewise diabetes, is associated with increase of cardiovascular diseases.

We found significant difference between type 1 diabetic patients and healthy controls in all values of 24 h Systolic (p < 0.001), 24 h Diastolic (p < 0.01), Day-time Systolic (p < 0.01), Night-time Systolic (p < 0.001), Day-time Diastolic (p < 0.05) and Night-time Diastolic (p < 0.001) blood pressure but only in night-time diastolic blood pressure values between diabetic patients and overweight subjects (p < 0.001).

Both type 1 diabetes and overweight are conditions with increased cardiovascular risk. Type 1 diabetes patients present in the long-term outcome microangiopathy and macro angiopathy such as nephropathy, retinopathy, and cardiovascular disease, that lead to increased morbidity and premature mortality [11] and elevated systemic blood pressure is a promoter of both the development and the progression of vascular sequelae such as diabetic kidney [12].

The pathogenesis of vascular complications still remains incompletely understood; however, endothelial dysfunction (ED) is thought to play a central role [13] and the mechanisms leading to ED in diabetes mellitus are multifactorial [14]. Large clinical trials have underscored the causative role of hyperglycemia, which contributes to ED by increasing production of reactive oxygen species and oxidative stress, activation of protein kinase C, and generation of vasoactive and proinflammatory substances. Increased oxidative stress is thought to be a central mechanism in the development of ED, and reactive oxygen species can directly disrupt the glycocalyx [15]. Interestingly, hyperglycemia and oxidative stress also trigger the production of advanced glycation end products, which exert potent proinflammatory activity. Elevated markers of inflammation such as C-reactive protein or interleukin-6 have been detected in children and adults with diabetes, [16, 17] supporting the notion that diabetes represents a condition of chronic inflammation.

There is some evidence that endothelial-dependent responses of the microvasculature and macro vasculature are impaired in children with type 1 diabetes when vascular complications are still subclinical [16, 18]. The prevention and early identification of vascular complications are a central issue in the care of patients with diabetes and the vigorous treatment of hypertension can be very effective in slowing the progression of diabetic kidney angiopathy and of other vascular diseases [19, 20].

In this prospective, the β2 adrenergic receptor seem play a role in both endothelial dysfunction and insulin release. Vascular β2 adrenergic receptors (β2ARs) mediate adrenergic vasorelaxation through direct activation of vascular smooth muscle cells, since the β2 ARs are expressed on endothelial cells (EC) and their stimulation causes endothelial nitric oxide synthase (eNOS) activation. This response is available to low concentration of agonist in several vascular districts [21].

The noradrenergic system provides fine-tuning to the endocrine pancreas activity through the function of a- and b-adrenergic receptors (ARs). The reciprocal regulation exerted by insulin and the adrenergic system has been well documented through a large number of studies. More recent evidence shows that mice with simultaneous deletion of the three known genes encoding the bARs (b1, b2, and b3) present a phenotype characterized by impaired glucose tolerance. Studies with b2AR agonists further suggest that the b2AR may play an important role in regulating insulin secretion. In addition, different human polymorphisms in the b2AR gene have been associated with higher fasting insulin levels. Nevertheless, the impact of the b2AR subtype on glucose tolerance and insulin secretion is still unclear [22].

Overweight and hypertension appear also to be inextricably linked [23]. The pathophysiology of obesity-related hypertension is complex, there are a large number of hypothetical mechanisms via which obesity is able to cause hypertension [24].

Among these, likewise to diabetes, there are also oxidative stress and inflammation that leads to endothelial dysfunction interacting with the microvascular endothelium in the mechanisms of obesity-associated hypertension [25]. On one hand infiltration and activation of macrophages in adipose tissue leads to chronic inflammation and secretion of inflammatory molecules, such as tumor necrosis factor-α, interleukin-6, monocyte chemoattractant protein-1, and inducible nitric oxide and on the other decreased nitric oxide impairs vascular relaxation leading to vasoconstriction for hypertension [26, 27]. Endothelial dysfunction and arterial stiffness are considered an independent predictor for the progression of cardiovascular events contributing to systolic hypertension [28, 29].

In our study we also observed a higher number of subjects with altered circadian blood pressure rhythm in type 1 diabetic patients and in overweight subjects than in healthy controls.

Lack of nocturnal fall in BP and higher nocturnal BP values suggest target organ damage in primary hypertension [30]. Obese adolescents have been already showed to present changes in blood pressure variability during 24-h in comparison with non-obese adolescents and for this reason they can early develop hypertension or other cardiovascular diseases in adult life [31].

Some cohort studies showed that blood pressure increases with increasing weight in both obese and normal weight individuals [32, 33]. In a study performed by Kotsis et al. the relationship between BMI and parameters resulted from 24-h ambulatory blood pressure monitoring of 3.216 hypertensive patients who were not on antihypertensive therapy showed that obese patients had increased ambulatory blood pressure parameters and altered circadian blood pressure rhythm with higher prevalence of non-dipping status [34].

There is some evidence that birth order influences growth and metabolism, from infancy to early adulthood. Importantly, first-born children have reduced insulin sensitivity and higher daytime blood pressure [35]. Although the height discrepancy is reduced by early adulthood, first-borns have greater adiposity. Further, first-borns have been shown to have a less favourable lipid profile in young adulthood, with higher LDL-C, total cholesterol, and triglyceride concentrations than later-borns. Thus, being firstborn may be associated with persistent changes in metabolism and body composition, that may lead to greater risk of developing type 2 diabetes mellitus and cardiovascular disease [35].

Some genes have been identified in the background of hypertension. CaMKIV plays a relevant role in the regulation of the vascular tone by a mechanism that involves eNOS activation through phosphorylative events. Impairment of CaMK-mediated activation of eNOS, as in CaMK4 gene deletion, induces hypertension, as demonstrated by the fact that CAMK4_/_ mice display a hypertensive phenotype that leads to typical organ damage [36].

The G-protein coupled receptor kinases (GRKs) are important regulators of beta-adrenergic signaling and play a role in regulation of peripheral vascular tone. Indeed, GRK2 levels and activity have been found increased in lymphocytes from hypertensive patients. Since a generalized impairment of b-adrenergic mediated vasodilation has been shown both in animal models of hypertension and in human hypertensive subjects, this alteration has been related to the increased GRK2 abundance and activity [37].

Down regulation of β-adrenergic signaling is mediated via GRK2 and GRK5 that phosphorylate cardiac β-adrenergic receptors leading to β-arrest in recruitment and G protein uncoupling. Given the important role of GRK2 and GRK5 in β-adrenergic signaling, functional genetic polymorphisms in the genes coding for GRK2 (*ADRBK1*) and GRK5 (*GRK5*) might be important pharmacogenetic targets [38].

Our study shows no significant differences in numbers of non-dippers among diabetics and overweight subjects, indicating that both the conditions present a greater risk for the alteration of the circadian rhythm of blood pressure and the hypertension than healthy control.

The non-dipper status, characterized by a decrease in physiological night-time drop of blood pressure, is the first sign of the increase of pressure load on blood vessels. This load is one of the factors leading to the vascular damage of the kidneys and eventually to diabetic nephropathy, being associated in type 1 diabetic patients with early morphologic changes in the glomerulus [39].

Alterations of the normal circadian blood pressure rhythm have been shown to be especially evident in micro- and macro albuminuric but are also more common in normoalbuminuric type 1 diabetic patients than in healthy controls, being related to the level of albumin excretion within the normoalbuminuric range and show a correlation with the progression of diabetic nephropathy and the development and progression of diabetic retinopathy [4, 13].

Perrin et al. evaluated serial biopsies in 29 normoalbuminuric type 1 diabetic patients and reported that 10 patients who developed microalbuminuria showed significant increases in their night time diastolic blood pressure and decreases in systolic and diastolic blood pressure dipping during 6 years of follow-up [40].

Although it is clear that microalbuminuric and hypertensive patients are subjects at risk, also clinically normotensive, normoalbuminuric patients with blunted circadian blood pressure variation may constitute another high-risk group [41].

Unlike to some previous studies, we did not find correlation between dipper status and the level of albumin excretion nor we found higher number of dipper status among patients with albuminuria. Similarly, in our study the alteration of circadian blood pressure rhythm did not seem related to duration of disease, HbA1c, Colesterol or BMI.

The pathogenesis of abnormal circadian blood pressure rhythm in diabetes is as yet unclear and several mechanisms have been proposed for the observed nighttime elevations in blood pressure. A role of latent overhydration [42] and possibly sodium retention [43] has been suggested, autonomic neuropathy has been implicated [44] and interactions between autonomic function, albumin excretion and blood pressure in the normoalbuminuric range have been previously described [42, 45]. Sympathetic over activation, implicated in non-diabetic kidney disease [46], has been shown to cause hyperfiltration in subjects with essential hypertension [47] and has been suggested as a feature of the nighttime increase in blood pressure in these patients [48]. Disturbances in parasympathetic function (the earliest phase of diabetic autonomic neuropathy)/are thought to be one of the causes of this sympathetic overactivity [49]. In addition to dysfunction of the sympathoadrenal system [50], the hypothalamo-pituitary-adrenal axis, the rennin-angiotensin-aldosterone system [51]and atrial natriuretic peptide [52], which normally also show circadian variations may also have a role.

Likewise, regarding non-dipper status in overweight subjects, similar mechanisms have been proposed. They include the activation of renin-angiotensin-aldosterone system in adipose tissue that lead to systemic vasoconstriction, sodium and water retention and aldosterone production, the alteration of sympathetic nervous system with a demonstrated higher increase of renal and cardiac norepinephrine excretion in overweight patients with hypertension (BMI >25 kg/m) compared with healthy, normotensive obese and metabolic dysregulation (including hyperinsulinemia that induces the activation of the SNS and also has a direct action on the kidney to stimulate sodium retention, adipokine imbalance, and increased inflammatory cytokines [53].

Angiotensin II promotes the growth and differentiation of adipocytes [54], the decrease in plasma adiponectin levels and increase of insulin resistance [55], induces the expression of C-reactive protein [56], and results in the secretion of leptin, which in turn activates the renal sympathetic nerve [57].

Attenuation of the nighttime fall in blood pressure has been shown to be associated with an increased risk of left ventricular hypertrophy, increased intima media thickness [58] and increased cardiovascular mortality [59].

Ambulatory blood pressure measurements in type 1 diabetic patients and overweight subjects should be considered during the follow-up of these patients because they can reveal an altered circadian blood pressure rhythm that has been shown to led to higher mortality and morbidity for vascular events as compared with patients with normal rhythms [60, 61].

Blood pressure exerts a dose-dependent effect on cardiovascular risk and it is involved in the developing of coronary artery disease [62].

The impact of sub clinical hypertension occurring at young ages is demonstrated by the correlations found between adolescent and adult BP readings. This relationship is illustrated by the ‘tracking’ phenomenon: subjects presenting BP values in a higher percentile during childhood and adolescence, likely will have their BP values in the same range during maturation, therefore it is possible to give a enough close prediction of who will have got a higher risk in adulthood [2]. Since adolescent BP readings are predictive of adult hypertension, early detection of the disease is very important. Based on data in the literature we can affirm that adolescent BP is related not only to hypertension but also to other cardiovascular diseases in adulthood [63]. Diagnosis of hypertension is closely dependent on accurate BP measurement. That is more important in adolescents as the misdiagnosis of hypertension may have a life-long negative impact on insurability and employment. ABPM gives several advantages in reaching high-quality BP determinations by avoiding observer errors [64].

### Limitations of the study

Our study has several limitations. This is a cross-sectional analysis, and therefore no data on the progression of complications or survival are available. The sample is relatively small, and thus additional studies in larger cohorts including longitudinal data are recommended to further study the prevalence of altered circadian blood pressure rhythm in type 1 diabetic patients.

Another limitation of our study is heterogeneity of age of the cohort, but this was necessary to assess a large-enough sample in this single-institution study.

We included only patients with type 1 diabetes who had relatively good control of their diabetes and thus these data cannot be generalized to those with poor control. However, the effect of poor control of diabetes on microvascular and macro vascular complications is already well known and has been studied in large multicenter trials. We did not subdivide or stratify by gender, so we could not study the difference between the sexes.

Future research in this area of inquiry should include the analysis of genetic profiles of diabetic, overweight and healthy controls, include larger samples drawn from diverse populations, stratify analysis by gender, to identify an eventual correlation between genetic features and clinical severity.

## Conclusions

Subclinical hypertension and reduced dipping can indicate incipient hypertension and ABPM studies might help to define a subset of patients at increased risk for the development of hypertension, who might benefit from the early introduction of anti-hypertensive strategies. We suggest to include ABPM in follow-up of type 1 diabetes and overweight subjects because it correlates much better with the prevalence of cardiovascular events than that provided by casual measurements. ABPM also offers numerous advantages over casual measurements in adolescents, as the measurements take place during everyday activities on the one hand and nighttime sleep on the other, thus providing continuous data about BP and it is better correlated with target organ damage than are office measurements. Considering the ‘tracking’ phenomenon in which adolescent hypertension translates into adult hypertension, it is of great importance that patients with the highest risks should be screened out based on reliable data.

## Abbreviations

ABPM: ambulatory blood pressure measurements
BP: blood pressure
HbA1c: glycosylated hemoglobin A1c
BMI: body mass index
ED: endothelial dysfunction
SD: Standard Deviation
OR: Odds Ratio
